# 肺癌伴子宫及附件转移1例并文献复习

**DOI:** 10.3779/j.issn.1009-3419.2024.102.40

**Published:** 2024-12-20

**Authors:** Jiangyan GUO, Pengju LUO, Mei LI, Jing YUAN, Meng CHEN, Shujie SONG

**Affiliations:** ^1^650118 昆明，昆明医科大学第三附属医院云南省肿瘤医院病理科; ^1^Department of Pathology, Yunnan Cancer Hospital, The Third Affiliated Hospital of Kunming Medical University,Kunming 650118, China; ^2^650041 昆明，昆明市第三人民医院综合内科; ^2^General Internal Department, The Third People's Hospital of Kunming, Kunming 650041, China

**Keywords:** 肺肿瘤, 子宫内膜肿瘤, 宫颈, 输卵管, 肿瘤转移, 免疫组化, Lung neoplasms, Endometrial neoplasms, Cervix, Fallopian tube, Neoplasm metastasis, Immunohistochemistry

## Abstract

原发性支气管肺癌简称肺癌，是世界各国发病率和死亡率较高的恶性肿瘤之一。 近年来，女性肺癌发病率升高且多为肺腺癌。因肺癌早期症状隐匿，发现时常伴有转移。对于原发性肺癌，子宫内膜及宫颈转移极为罕见。本文回顾性分析1例肺微乳头亚型腺癌伴子宫及附件转移患者的临床病理资料，并通过形态、免疫组化及分子检测进行证实，由此为肺癌伴子宫及附件转移的临床管理提供参考。

## Abstract (en)

Primary bronchial lung cancer, commonly known as lung cancer, is one of the malignant tumors with high morbidity and mortality in the world. In recent years, the incidence of lung cancer in women has increased, and most of them are lung adenocarcinoma. Because the early symptoms of lung cancer are occult, it is often detected when matastasis has already occurred. Endometrial and cervical metastasis is extremely rare for primary lung cancer. This article retrospectively analyzed the clinicopathological data of a patient with pulmonary microcapillary subtype adenocarcinoma with uterine and adnexal metastasis, and confirmed by morphology, immunohistochemistry and molecular detection, in order to provide references for clinical management of uterine and adnexal metastasis of lung adenocarcinoma.

据2023年癌症统计数据^[1]^显示，肺癌的新发病率位于第一位，乳腺癌位于第二位。肺癌死亡率仍在首位，是癌症死亡的主要原因。女性肺癌的发病率逐年增高^[2]^ ，不少患者首次就诊时已出现转移，区域淋巴结、肝脏、肾上腺、骨骼和脑是肺腺癌最常见的转移部位。肺癌转移至女性生殖道的情况很少见。卵巢和阴道受累是外生殖器和生殖器原发性恶性肿瘤最常见的妇科转移部位^[3]^，卵巢是原发性肺腺癌相对常见的转移部位^[4]^。本病例以异常阴道出血就诊，最终诊断为肺腺癌，微乳头亚型（micropapillary predominant adenocarcinoma, MPA）伴子宫内膜及附件转移，现报告如下。

## 1 病例资料

患者，女性，47岁，2016年曾出现经期大量流血，流血不止，至当地医院就诊，经诊刮后好转；2020年4月无明显诱因出现腹胀，无腹痛，当地医院B超提示子宫肌瘤，嘱定期复查。2021年5月下腹部可扪及包块，未就诊。2022年2月子宫增大平脐，如孕5月余。2022年3月无明显诱因突然出现阴道大量流血，色鲜红伴大量血凝块，流血不止伴腹痛，头晕无力，120送至当地医院给予输血治疗后好转，后转至妇科行诊断性刮宫，探针无法进入宫腔，建议促性腺激素释放激素激动剂（gonadotropin-releasing hormone agonist, GnRH-a）治疗3个周期后手术，后因当地医院麻醉科评估无法手术，建议至上级医院就诊。患者2022年4月至云南省肿瘤医院就诊，行正电子发射计算机断层扫描（positron emission tomography/computed tomography, PET/CT）检查（图1）：（1）子宫体明显增大并不规则软组织肿块形成，大小约14.9 cm×15.5 cm×16.6 cm伴代谢增高，考虑恶性病变，子宫内膜癌可能性大；（2）中下腹腹膜后、双侧髂血管旁多发代谢增高淋巴结，考虑转移；双侧卵巢显示不清；（3）左肺上叶舌段不规则团片状影伴代谢增高，大小约4.3 cm×2.0 cm，边缘毛糙并多发长条索，粘连牵拉胸膜，周围见斑片状磨玻璃样密度影，考虑肺癌可能性大；（4）前纵隔区、纵隔4R组、双肺门区、双侧锁骨上区、左侧腋窝代谢增高淋巴结，考虑转移；（5）全身多处骨骼多发不同程度代谢增高灶。血肿瘤标志物检测：细胞角蛋白19片段（cytokeratin-19 fragment, CYFRA21-1）水平升高（92.60 ng/mL），癌胚抗原（carcinoembryonic antigen, CEA）水平升高（34.80 μg/L），糖类抗原72-4（carbohydrate antigen 72-4, CA72-4）水平升高（28.10 KU/L），CA15-3水平升高（70.10 KU/L）。2022年4月25日至我院妇科行锁骨上淋巴结穿刺，病检提示左右锁骨上淋巴结薄层液基细胞片均检出癌细胞。宫腔刮出物为低分化腺癌，具体分型请待术后进一步诊断。完善相关检查，排除手术禁忌，于2022年4月29日全麻下行次广泛全子宫+双侧附件切除术+盆腔淋巴结清扫术。术后病理为低分化腺癌（图2A-2E），支持肺来源。左髂总淋巴结9枚、左腹主淋巴结9枚、左盆腔淋巴结9枚、右髂总淋巴结3枚、右腹主淋巴结8枚、右盆腔淋巴结7枚、左肾门肿大淋巴结14枚、骶前淋巴结1枚镜下均为肺腺癌转移。免疫组化：肿瘤细胞表达：细胞角蛋白7（cytokeratin 7, CK7）阳性（图2G），甲状腺转录因子-1（thyroid transcription factor-1, TTF-1）阳性（图2H），新天冬氨酸蛋白酶A（Napsin A）阳性（图2I），表面活性蛋白（surfactant protein B, SPB）阳性（图2J）；雌激素受体（estrogen receptor, ER）阴性，孕激素受体（progesterone receptor, PR）阴性，P53阴性、P16阴性、波形蛋白（Vimentin, Vim）阴性，配对盒基因8（paired box gene 8, PAX-8）阴性（图2K），肾母细胞瘤基因1（Wilms tumor gene 1, WT-1）阴性（图2L），绒毛蛋白（Villin）阴性，尾型同源框基因2（cadual type homeobox gene 2, CDX2）阴性（图2M），细胞角蛋白20（cytokeratin 20, CK20）阴性。肿瘤细胞Ki-67增殖指数约10%。术后，病情平稳后，于2022年6月1日行左肺部肿物穿刺。病理提示肺腺癌，见微乳头亚型（图2F）。6月3日行TP方案化疗。肺肿瘤168基因检测结果：表皮生长因子受体（epidermal growth factor receptor, EGFR）外显子19框内缺失 突变。子宫肿块EGFR基因检测：EGFR外显子19框内缺失突变。分子检测EGFR外显子19突变，有靶向治疗指征，口服埃克替尼治疗，目前已治疗5个周期。患者术后恢复良好，靶向治疗疗效评效疾病稳定，继续口服靶向药，随访至今22个月，未持续进展。

**图 1:**
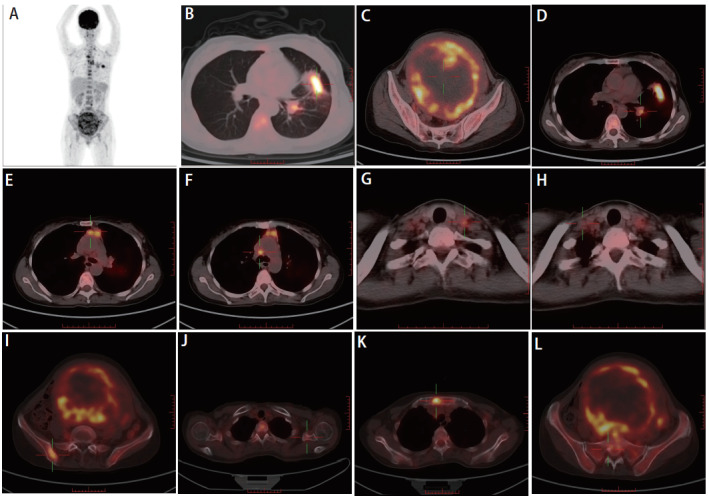
患者病灶的PET/CT影像学资料。A：全景图；B：左肺上叶舌段肿块；C：子宫肿块；D：左肺门区淋巴结转移；E：前纵隔区淋巴结转移；F：纵隔4R区淋巴结转移；G：左腋窝淋巴结转移；H：右锁骨区淋巴结转移；I：右侧髂血管淋巴结转移；J：左侧肩胛骨转移；K：胸骨转移；L：骶骨转移。

**图 2:**
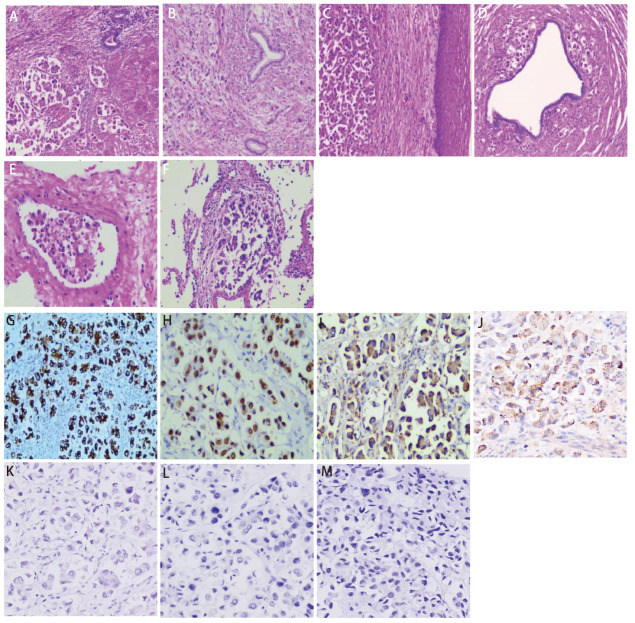
肿瘤HE染色（×100）及免疫组化结果（EnVision, ×200）。HE染色：A：子宫内膜；B：宫颈内口；C：宫颈外口；D：输卵管；E：癌栓；F：肺部肿块穿刺；免疫组化：子宫内膜CK7（G）、TTF-1（H）、Napsin A（I）及SPB（J）阳性表达，Pax-8（K）、WT-1（L）及CDX2（M）阴性表达。

## 2 讨论

肺癌是全球癌症相关死亡的主要原因，尤其是非小细胞肺癌（non-small cell lung cancer, NSCLC），不少患者就诊时已出现转移。NSCLC转移的主要部位包括脑（47%）、骨（36%）、肝脏（22%）、肾上腺（15%）、胸腔（11%）和远处淋巴结（10%）^[5]^。其他器官转移非常罕见，一般不到5%。Mazur等^[3]^研究发现，在149例女性生殖系统转移性肿瘤患者中，卵巢和阴道是外生殖器和生殖器原发灶最常见的转移部位，卵巢约占75.8%，阴道约占13.4%，少见于子宫，仅占8.1%。肺腺癌极少转移至子宫的原因可能由于子宫器官较小，减少了远端血液在其内流通以及其含有较多纤维和平滑肌组织，不利于恶性肿瘤的播散。查阅国内外文献报道与肺癌发生子宫转移密切相关的文献近十余篇 ，如表1显示^[6⇓⇓⇓⇓⇓⇓⇓⇓⇓-16]^。

**表 1:** 肺腺癌子宫内膜和附件转移的既往病例

Author	Age (yr)	The first symptom	Histological type	Metastatic	Histological finding	EGFR mutation	Stage when first dignosed
Chen YX, et al^[6]^	50	Abnormal vaginal bleeding	Lung adenocarcinoma	Endometrial	CK7, TTF-1 and Napsin A positive	No detection	IV
Bulutay P, et al^[7]^	83	Postmenopausal uterine bleeding and anemia	Lung adenocarcinoma	Endometrial	TTF-1, Napsin A positive; Pax-8 negative	Exon 19	IV
Xu L, et al^[8]^	63	Cough lasting for one month	Lung adenocarcinoma	Cervical	CK7, TTF-1 positive	Exon 21 p.L858R	IV
Wang Y, et al^[9]^	49	Lumbago and sacroiliac joint pain; Vajinal bleeding	Lung adenocarcinoma	Cervical	CK7, TTF-1 and Napsin A positive	Exon 19	IVB
Knox B, et al^[10]^	65	A flurodeoxyglucoseavid uterine lesion, following excision of a local lung adenocarcinoma	Lung adenocarcinoma	Uterus	CK7, TTF-1 and Napsin A positive	No detection	IV
Chen KL, et al^[11]^	45	Intermittent cough and sputum with time for six months	Lung solid mucinous cell adenocarcinoma	Endometrial	CK7, TTF-1 and Napsin A positive	No detecion	IV
Shibata M, et al^[12]^	63	Abnormal vaginal bleeding	Lung adenocarcinoma	Uterine	No detection	Exon 19	IV
Ahmad Z, et al^[13]^	51	Abnormal genital bleeding	Lung adenocarcinoma	Uterus	CK7, TTF-1, Napsin A and MOC31 positive	Exon 21 p.L858R	IV
Kajimoto N, et al^[14]^	82	Abnormal genital bleeding	Lung adenocarcinoma	Endometrial	CK7, TTF-1 and Napsin A positive	Exon 21 p.L858R	IV
Kentaro K, et al^[15]^	69	Upper lobectomy for lung cancer; General fatigue and atypical gential bleeding	Lung adenocarcinoma	Cervical	CK7, TTF-1 and Napsin A positive	No detection	IIIB
Chargari C, et al^[16]^	56	Invasive ductal carcinoma of the breast, stage T2, found during follow-up examination	Small cell carcinoma	Uterus	CK7, TTF-1, Syn, CgA and Ki-67 (>60%) positive	No detection	IV

EGFR: epidermal growth factor receptor.

从表1中我们发现，形态学上，子宫内膜转移性病变很难与原发子宫恶性肿瘤区分，需借助免疫组化染色，如TTF-1是甲状腺和肺上皮发育过程中产生的组织特异性转录因子，TTF-1核表达是区分原发性肺腺癌和其他部位腺癌的最敏感和最具特异性的标志物^[17]^。74%-92%的肺腺癌病例表现出TTF-1核表达^[18]^，而仅6%-32%的子宫内膜腺癌中表达TTF-1^[19]^。几乎100%被诊断为子宫内膜腺癌的患者和90%的肺腺癌患者为CK7阳性和CK20阴性^[20]^。因此，TTF-1阳性、CK7阳性和CK20阴性免疫表型的组合表达高度提示肺原发性腺癌（特异性100%）^[21]^。Napsin A是1998年被Tatnell等^[22]^发现的一种显著表达于肺和肾脏的天门冬氨酸蛋白酶，鉴别价值及特异性均优于TTF-1^[23]^。SFTPB基因^[24]^仅在肺和胎肺中表达，在NSCLC发生发展过程中发挥重要功能，编码肺泡SPB，诊断肺腺癌的特异性为90.7%，敏感性为43.7%。结合本例，宫腔病灶免疫组化CK7阳性、TTF-1阳性、Napsin A阳性及SPB阳性，提供了肺部来源的证据。

为了寻找更多的证据，在本例中，患者病情平稳后，进行肺穿刺，穿刺后的形态与子宫及附件的形态一致，肺泡腔内见单个、多个簇状肿瘤细胞，无纤维血管轴心，为NSCLC，肺腺癌，经典型MPA。MPA是具有高度侵袭性的一种亚型，常有血管、淋巴管和间质侵犯，有时可见砂粒体，与其他亚型相比，恶性程度高，复发率高，更易发生淋巴结转移及远处转移，预后差^[25]^，与本例子宫转移、盆腔淋巴结转移及大量脉管内癌栓相呼应。因此子宫、附件的病灶是肺腺癌的继发灶，而非原发^[26]^。

分子检测除了可以帮助患者寻找靶点基因，在确定肿瘤的起源上也具有一定提示作用。EGFR基因突变在肺腺癌中多见，但在其他器官的癌症中很少见，多发生于无吸烟习惯、亚洲女性和腺癌患者。EGFR突变可能与NSCLC更具侵袭性的肿瘤进展有关。因此，EGFR突变的女性肺腺癌可能促进生殖系统转移^[8]^。据报道^[27]^EGFR突变的位点与NSCLC的不同远处转移有关。EGFR外显子19突变与脑转移更相关，EGFR外显子21 p.L858R突变与肝转移更相关^[28]^。EGFR突变的NSCLC中的子宫转移可能与特定的因素相关，但需更多样本来验证这一点。本例中，原发灶及转移灶都出现了EGFR外显子19的突变，从分子的角度也支持肺原发，而非生殖系统原发。

在治疗方面，肺转移至子宫十分罕见，对于转移灶是否切除尚无统一标准，即使盆腔肿瘤切除可能有助于缩小肿瘤体积，减轻肿瘤负荷，但目前尚无证据可以延缓患者的生存期，改善患者的预后^[29]^。如果更早被识别为转移性病变，可以通过早期的局部治疗来缓解。

本病例报道中患者从最初不被重视的症状到确诊，肿瘤已广泛播散及转移，若能及时对宫腔出血引起警惕，及时排查，让患者早诊断、早治疗，患者的治疗结局将大大不同。同时也警醒临床医生，以异常阴道出血为首发症状且伴有下腹包块患者就诊时，PET/CT检查可有助于评估肿瘤的转移情况。临床病史、影像学资料、病理形态、免疫组化及分子突变分析可共同确定肿瘤是原发或转移，为后续临床制定最佳治疗策略奠定基础。
